# Sex and gender differences in social participation among community-dwelling older adults: a systematic review

**DOI:** 10.3389/fpubh.2024.1335692

**Published:** 2024-04-12

**Authors:** Chuan Hong Ong, Bang Linh Pham, Mélanie Levasseur, Guang Rong Tan, Betsy Seah

**Affiliations:** ^1^Nursing Service, Tan Tock Seng Hospital, Singapore, Singapore; ^2^Nursing Service, National University Hospital, Singapore, Singapore; ^3^School of Rehabilitation, Faculty of Medicine and Health Sciences, Université de Sherbrooke, Sherbrooke, QC, Canada; ^4^Alice Lee Centre for Nursing Studies, Yong Loo Lin School of Medicine, National University of Singapore, Singapore, Singapore

**Keywords:** aged, community, sex differences, social participation, social prescription, systematic review

## Abstract

**Background:**

Frequent social participation among older adults is associated with greater health. Although understanding how sex and gender influence social participation is important, particularly in developing sex-inclusive health promotion and preventive interventions, little is known about factors influencing engagement of older women and men in social activities.

**Aim:**

This study thus aimed to examine factors influencing social activities of older women and men.

**Methods:**

A mixed-method systematic review was conducted in nine electronic databases from inception to March 2023. The studies had to define social participation as activities with others and examine its influencing factors among community-dwelling older women and men. Data were analyzed using convergent synthesis design from a socio-ecological perspective.

**Results:**

Forty-nine studies, comprising 42 quantitative, five qualitative and two mixed method design were included. Themes identified concerned: (a) sociodemographic factors, (b) personal assets, (c) interpersonal relationships and commitments, (d) physical environment, and (e) societal norms and gender expectations. The findings identified the heterogeneous needs, preferences and inequalities faced by older women and men, considerations on sociocultural expectations and norms of each gender when engaging in social activities, and the importance of having adequate and accessible social spaces. Overall, this review identified more evidence on factors influencing social participation among women than in men.

**Conclusion:**

Special attention is needed among community care providers and healthcare professionals to co-design, implement or prescribe a combination of sex and gender-specific and neutral activities that interest both older women and men. Intersectoral collaborative actions, including public health advocates, gerontologists, policymakers, and land use planners, are needed to unify efforts to foster social inclusion by creating an age-friendly and sustainable healthy environment. More longitudinal studies are required to better understand social participation trajectories from a sex and gender perspective and identify factors influencing it.

**Systematic reviews registration:**

http://www.crd.york.ac.uk/PROSPERO, identifier [CRD42023392764].

## 1 Introduction

Worldwide, the proportion of people aged 60 and older is expected to more than double in the next 30 years and surpass 1.5 billion by 2050 (1). Over their life course, people often lose their social roles than acquire new ones (2, 3) but, as one ages, they seek for continuity in social relationships and engagement in social activities in the community (4). Social participation has been broadly described as an individual’s involvement in activities that provide opportunities for connection with others in community life and other important shared areas (5). Changing in response to available time and resources and based on societal context and what individuals perceived as meaningful (5), social participation has steadily declined, giving rise to a new epidemic of loneliness and isolation (6). Frequent social participation among older adults contributes to greater social integration and improves health outcomes such as cardiovascular health (7–9) and cognition (10). Greater social participation also reduces the risk of social isolation, increases socioemotional support, promotes a sense of being valued (7, 11), and protects against depressive symptoms (12, 13). Given the potential benefits of social participation, a substantial body of literature and reviews have examined the associations between social participation and various health outcomes (10, 14). Studies have also examined the effectiveness of various interventions fostering social participation, including the use of information and communication technologies (ICTs), among older adults (15–18). A recent study by Alvarado Vazquez, Madureira (19) highlighted the potential of utilizing ICTs in the planning, design, and maintenance of public spaces to enhance social participation. In their chapter within the book “Aging, Technology and Health”, Bixter, Blocker (20) discussed the potential that social engagement technologies hold to alleviate the barriers to social interactions that numerous older adults encounter, including physical, cognitive, and financial obstacles. As poorer health outcomes have been found to be associated with social isolation among older adults, it is imperative to accelerate the development of interest, and building of capabilities and competencies for social prescription programs linked to community resources and fostering social participation (21, 22). Although the global benefits of social participation in old age are obvious (23, 24), differences in social participation have been found according to sex and gender (25, 26).

Usually categorized as male or female, sex refers to biological characteristics associated with physical and physiological features, while gender is socially constructed based on roles, behaviors, expressions, and identities of women, men, and gender-diverse people (27). Growing older is not the same for everyone. Across one’s lifespan, sex and gender influence social opportunities and economic means, which are linked to healthy lifestyle choices (28), including social participation. Among the several studies that have examined social participation among community-dwelling older adults, some have identified specific sex and gender differences. However, the existing literature lacks a comprehensive understanding of the factors influencing the engagement in social activities between older women and men. Personal factors include health, resilience, and personality. For example, participation in social activities is more likely to be affected by poor health in older men than women (29). Furthermore, physical and social characteristics of the environment can influence social participation differently according to sex. For instance, one study using photography (30) reported that most interactions within open public spaces were segregated by sex. Moreover, differences between genders may be influenced by sociocultural factors. In Chinese culture, Confucianism is a significant social value that requires women to be responsible for the household and men to work as breadwinners (31). Older women were thus confined to primarily caring for the family and less likely to develop close relationships with friends (32).

To our knowledge, a rigorous, integrative, and comprehensive portrait of social participation in older adults specific to sex and gender and the underlying factors that influence it is still lacking. While systematic reviews have examined social participation according to age (33) and its barriers and facilitators among older adults (34, 35), knowledge specific to sex is limited. Therefore, conducting a mixed method systematic review is necessary to address this gap in the literature and comprehensively synthesize the existing evidence to inform healthcare professionals, community care providers, and policymakers on ways to improve social participation from a sex and gender perspective. This study thus aimed to provide a comprehensive understanding of factors influencing social participation according to sex and gender among older adults. Such a synthesis of current knowledge represents an original contribution and may ultimately support decisions and the development of innovative policies and practices improving social participation among all older adults.

## 2 Methods

Following Pluye and Hong (36) framework using a convergent integrated approach, the review was driven by a broad question: What are the factors influencing older women and men when engaging in social participation? The protocol was registered in the International Prospective Register of Systematic Reviews (PROSPERO) (registration number CRD42023392764). This study adhered to the Preferred Reporting Items for Systematic Reviews and Meta Analyses (PRISMA) guidelines (37) (Appendix A).

### 2.1 Eligibility criteria

This review included studies involving older adults aged 60 and older (Table 1) who were community-dwelling or living in residential settings (38). Studies that focused on specific clinical populations were excluded (e.g., individuals with knee osteoarthritis, suicidal ideation, Alzheimer’s disease). While this review acknowledged the complexity of gender and that sex and gender have distinct meanings, a binary conceptualization had to be used where individuals were identified as male or female. This conceptualization is justified by the confusion surrounding “gender” and “sex” where the majority of the studies used these terms interchangeably and presented results according to sex only and not gender or did not fully consider the extent of gender. Studies reporting no specific sex or gender-related findings were excluded.

**Table 1:** Taxonomy of social activities.

Level	Definition (Levasseur et al., 2010)
1	“Doing an activity in preparation for connecting with others” includes both activities of daily living and instrumental activities of daily living.
2	“Being with others (alone but with people around),” like walking in the community.
3	“Interacting with others (social contact) without doing a specific activity with them,” like shopping.
4	“Doing an activity with others (collaborating to reach the same goal)” meant individual collaborates with others to perform an activity and reach a common goal.
5	“Helping others,” such as being a caregiver or volunteer.
6	“Contributing to society” included individual contributes broadly to civic activities.

To distinguish social participation from parallel but different concepts, the 6-level Taxonomy of social activities (39) was used (Table 2). This review only included studies that defined social participation as activities performed with others, which refers to level three to level six of the taxonomy. These social activities can be performed with or without a common goal or benefiting others or society. Studies were thus excluded when (i) if social activities examined lacked interaction with other people, (ii) it was unclear if the studies focused on activities involving interaction with others, and (iii) focused on a single type of social activity (e.g., only volunteering, caregiving, and religious participation), which reflected too narrow participation (40). To comprehensively inform about the influence of sex and gender-specific factors on social participation among older adults, this review included quantitative, qualitative, and mixed methods studies (Table 1). Only studies that were available in full texts were included in the data synthesis of this review.

**Table 2:** Eligibility criteria of studies.

PICOS	Inclusion criteria	Exclusion criteria
Population	Older adults aged 60 years old and above	Specific clinical populations
Interest	Studies that examine gender difference in social participation (level 3–6 of the taxonomy of social activities)	Measurement of social participation that included all levels of the taxonomy or included social activities involving level 1–2 only.
Context	Living in residential settings in the community	Inpatient or living in aged care facilities
Outcomes	Studies that highlight the factors (facilitators or barriers) influencing social participation in older men and women	Incidental findings (Findings outside of the study aim/s)
Study designs	Quantitative, qualitative, mixed methods study designs	Systematic review, scoping review, literature review, concept analysis papers, editorials, discussion papers

### 2.2 Search strategy

A three-step search strategy was used. An initial search limited to PubMed and Scopus was first carried out. The words in the title and abstracts of relevant articles and index terms were used to develop a full search strategy refined by a university research librarian (Appendix B). Keywords included were related to ‘older adults,’ ‘social participation,’ and ‘sex/gender differences.’ ASSIA, CINAHL, Embase, PsycINFO, PubMed, Scopus, Social Science Database, Web of Science, and ProQuest Theses and Dissertations were searched for studies published from inception to March 2023. The search was limited to ‘humans’ and ‘English language’ articles but without restrictions to publication date or geographic area. The inclusion of English publications only ensures consistency and coherence of the process by simplifying the synthesis and interpretation of data and reducing potential linguistic and translational barriers that might arise when dealing with multiple languages. Reference lists of included articles were manually searched to identify additional relevant studies.

### 2.3 Study selection

With studies imported into EndNote Version 20 (41), two independent reviewers (OCH & PBL) screened all the titles, abstracts, and full texts of potential studies. Any disagreements were resolved through discussion, with the assistance of a third reviewer (BS).

### 2.4 Assessment of methodological quality

Two reviewers used five tools to independently assess the methodological quality according to the design (OCH and PBL). Joanna Briggs Institute (JBI) Checklists (42) were used for cross-sectional, cohort, and prevalent studies, while Critical Appraisal Skills Programme (43) and Mixed Methods Appraisal Tool (44) were used, respectively, for qualitative and mixed methods studies. Discrepancies were resolved through discussion between the two reviewers. As recommended by Hong, Pluye (44), no study was excluded based on quality appraisal to consolidate all available evidence and provide insights on sex and gender differences in social participation.

### 2.5 Data extraction

The following data were extracted by OCH: studies’ characteristics (author, publication year, country, other context-related information) and other descriptive information (aim, design, sampling method, participant characteristic, the phenomenon of interest, reported findings, and text relevant to our research objective, emergent themes, authors’ conclusions). These data were recorded in an extraction form modified from the JBI manual, which was pilot tested to ensure reliability, i.e., completed by both reviewers, compared, and improved after discussion. The data extracted was independently reviewed by PBL. All disagreements were resolved through discussions between the two reviewers. Although authors of seven papers were contacted to request for missing or additional data and four of them responded, the information provided was either akin to the published data or limited and could not be used.

### 2.6 Data transformation and synthesis

The use of the advanced qualitative convergent meta-integration was justified by the review question, in which identifying influencing factors predispose to include work being qualitative in nature and mixed methods studies (45). First, the studies were categorized, and mixed methods studies were fractionated into qualitative and quantitative data and evidence. Data transformation was performed to ensure that included studies were analyzed using the same synthesis method. Quantitative data and evidence were then narratively summarized, as recommended in the JBI manual (46). Next, iterative intra-method analysis and synthesis were conducted; the transformed quantitative and qualitative datasets were coded separately, with emerging findings compared. This step was followed by iterative inter-method integration; the two sets of codes were integrated and compared. Finally, ‘qualitized’ and qualitative findings were integrated using thematic synthesis (47) based on the socio-ecological model (48). This model was used to comprehensively understand the socio-ecological context that influenced social participation among older women and men across different domains, including individual, interpersonal relationships, community, and societal levels. Line-by-line inductive coding allowed the first author (OCH) to create codes, which were then deductively matched and grouped into categories to identify specific descriptive factors influencing social participation. These factors were identified and synthesized by re-examining the inferred evidence with the studies’ textual data, generating themes. Finally, the descriptive factors and themes were finalized when consensus was reached through discussions among the research team.

## 3 Results

**Figure 1:**
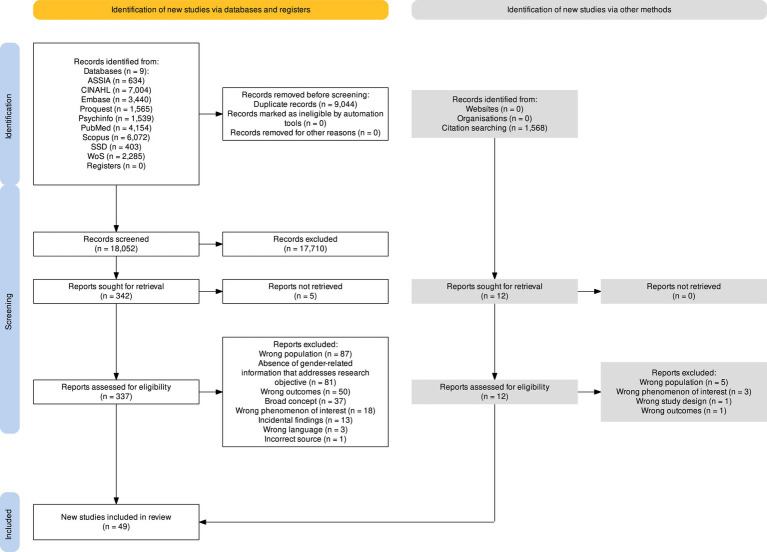
PRISMA flow diagram (59).

A total of 27,096 records were retrieved. After deduplication, 17,709 papers were screened for their title and abstracts and 343 full texts (Figure 1). After adding two studies from reference lists, 49 studies were subjected to quality appraisal, data extraction, and analysis.

### 3.1 Study characteristics

Twenty studies were conducted in Asian countries, 12 in North America, eight in Europe, three in South America, two in Middle Eastern, three in Australia, and one in multiple European countries (Table 3). The study designs varied: 42 were quantitative, five were qualitative, and two mixed methods. Approximately two-thirds (*n* = 34; 69.4%) of the studies were published within the last decade (2012–2023).

**Table 3:** Characteristics of included studies.

Author, year, country	Study design	Study sample (number, age, gender)	Data collection methods	Social participation (definition/measure)
Adamson, 2004, United Kingdom	Cross-sectional	4,286 Aged 60–79 Female only	Questionnaire, interview, physical examination, and blood sampling	WHO definition of participation restriction Table 1 (types of social activities)
Amirkhosravi, 2015, Iran	Cross-sectional	525 Above 60 Both genders	Three-part instrument. If illiterate, instrument completed by researcher through interviewing	Social participation questionnaire (consists of 17 items in two components of formal and informal social participation)
Ang, 2018, Singapore	Cross-sectional	4,482 Above 60 Both genders	Self-report	Assessed through self-reported engagement in five social activities
Atchley, 1975	Cross-sectional	902 Aged 70–79 Both genders	Mail questionnaires	
Bukov, 2002, Germany	Cohort study	516 at T1 and 206 at T3 Above 70 Both genders	Berlin Aging Study (interview)	11 activity domains (hobby, traveling, day trips, sports, culture, games, education, art, dancing, voluntary social engagement, and politics)
Costa, 2019, Brazil	Cross-sectional	2,344 Aged 72.3 ± 5.5 Both genders	FIBRA study Data collection session held in easily accessible public places	Evaluated through a 13-item inventory (AAVD)
Choi, 2021, South Korea	Cross-sectional	4,608 Above 65 Both genders	Living Profiles of Older People Survey (LPOPS) conducted by Korean Ministry of Health and Welfare	Frequency of participation in seven types: (a) attendance of alumni meetings, (b) religious meetings, (c) social clubs (in a senior center), (d) sports club, (e) lifelong education classes, (f) voluntary activities, (g) community welfare center activities
Dury, 2021, Belgium	Cohort study	372 Above 60 Both genders	Interview-based survey	Applied the classification system of Levasseur et al. (2010) differentiating the six distal to proximal levels of involvement with others in social activities.
Eckert, 1985, United States	Cross-sectional	694 Above 60 Both genders	Interview survey, supplemented by qualitative data gathered during onsite observations and interviewing, local census and agency information and published studies on aging.	Nine measures fall into three groups – (a) measures of general social involvement, (b) of involvement with age peers, (c) of help channel choices
Giesel, 2015, Germany	Mixed methods	771 Above 65 Both genders	Germany-wide mobility survey	
Goto, 2022, Japan	Cross-sectional	1,301 Aged 65–85 Both genders	Mail questionnaires	Addressed by the following question: “Do you participate in any community activities or volunteer activities?”
He, 2017, China	Cross-sectional	2,644 Above 60 Both genders	Structured interview	Measured by several questions regarding their participation in five categories of social activities over the past month.
Hsu, 2019, Taiwan	Cross-sectional	738 Above 55 (age stratified) Both genders	Face to face survey	Five indicators were used to measure social participation: (a) volunteering in the past 12 months, (b) caring for children or grandchildren at least once per week, (c) caring for older adult or disabled family members at least once per week, (d) political participation in the past 12 months, (e) participation in other social groups at least once per month
Isherwood, 2017, Australia	Qualitative	20 Aged 85–96 Both genders	In-depth semi-structured interviews	Participants were asked how they typically spent their time, including their social participation and activities in the home.
Isherwood, 2023, Australia	Qualitative	20, Aged 85–96 Both genders	In-depth semi-structured interviews	
Katagiri, 2018, South Korea and Japan	Cross-sectional	683 Japan, 362 Korea Above 65 Both genders	Face to face interviews	Participation in seven types of activities: (a) political associations, (b) residential/neighborhood associations, (c) social service clubs, (d) citizen movement/consumer cooperative groups, (e) religious groups, (f) alumni associations, (g) recreational associations.
Katja, 2014, Finland	Cross-sectional	1,181 Aged 65–84 Both genders	Face to face interviews	Collective social activity – asked about their involvement in different kinds of hobbies. Productive social activity – asked about giving help to relatives, friends, or neighbors
Khadr, 2011, Egypt	Cross-sectional	867 Above 60 Both genders	Survey	Social activities: (a) entertaining children through storytelling and other activities, (b) discussing important family and community historical events, (c) helping children with their school work, (d) teaching vocational and craft skills, (e) providing emotional support, (f) negotiating and advising matrimonial arrangements, (g) serving as mediator in conflict, (h) provide advice for traditional medications and healing
Kim, 2017, United States	Cross-sectional	6,476 Above 65 Both genders	National Health and Aging Trends Study (NHATS)	Informal social participation: (a) visit in-person with friends or family not living with sample person, (b) going out for enjoyment. Formal social participation: (a) attending classes, clubs, or organized activities, (b) volunteering, (c) religious participation
Lee, 2019, South Korea	Cohort study	3,729 Aged 55–84 Both genders	Korean Longitudinal Study of Aging (KLoSA)	Types of socially productive activities: (a) church or other religious gatherings, (b) friendship organizations, (c) alumni associations, (d) volunteering
Lee, 2020, South Korea	Cohort study	2,573 Above 60 Both genders	Korean Longitudinal Study of Aging (KLoSA)	Activity participation was considered under two broad domains: social and labor participation
Levasseur, 2011, Canada	Cross-sectional	554 Aged 68–82 Both genders	Survey	Social portion of the “Elderly Activity Inventory Questionnaire” and Statistics Canada’s Participation and Activity Limitation Survey adapted to assess social participation
Levasseur, 2017, Canada	Cross-sectional	4,541 Aged 60–106 Both genders	Phone interview	Statistics Canada’s Participation and Activity Limitation Survey
Li, 2011, Taiwan	Cross-sectional	220 Above 65 Both genders	Interview survey	Social activity questionnaire
Li, 2014, China	Cross-sectional	10,016 Above 60 Both genders	Survey	Productive activities – paid work, providing assistance to family, volunteering
Lu, 2018, China	Cross-sectional	456 Above 60 Both genders	Face to face interviews	Structural community social capital was assessed by the number of membership organizations, social participation and citizenship activities.
Marsh, 2018, Sri Lanka	Mixed methods	1,200 (1,028 for analysis) Above 60 Both genders	Survey and focus group discussions	Frequency of participation in eight organized social activities
Martinez, 2009, United States	Qualitative	35 Aged 61–87 Both genders	Focus group discussions	Level of participation on 12 social activities derived from an existing questionnaire
Naud, 2019, Canada	Cross-sectional	16,274 Aged 65–104 Both genders	Face to face interviews	Frequency of participation in eight community activities: (a) family or friends outside the household, (b) church or religious, (c) sports or physical, (d) educational and cultural, (e) service club or fraternal organization, (f) neighborhood, community or professional association, (g) volunteering or charity work, (h) other recreational
Naud, 2021, Canada	Cross-sectional	30,865 Above 45 (age stratified) Both genders	Face to face interviews	Frequency of participating in eight social and community activities: (a) family or friends outside the household, (b) church or religious, (c) sports or physical, (d) educational and cultural, (e) service club or fraternal organization, (f) neighborhood, community or professional association, (g) volunteering or charity work, (h) other recreational
Park, 2010, South Korea	Cross-sectional	761 Aged 65–84 Both genders	Survey	Participation in at least one of the following social activities: (a) job activities, (b) religious gatherings, (c) volunteer service
Park, 2013, United States	Cross-sectional	674 Above 60 Both genders	Survey questionnaires	Three social engagement-related variables were used – (a) living arrangement, (b) social network, (c) Participation in social activities
Ponce, 2014, Chile	Cross-sectional	31,428 Above 60 Both genders	National Socioeconomic Characterization survey (CASEN) conducted by Chilean Ministry of Social Development	For assessing the variable social participation, following question was asked: “Are you currently involved in an association or organized group?”
Rozanova, 2012, Canada	Interpretive qualitative	89 Above 65 Both genders	Semi-structured 60–90-min-long qualitative interviews	
Sabbath, 2016, France	Prospective cohort study	10,764 Aged 60–74 Both genders	Self-report questionnaire GAZEL	Active life engagement: (a) paid work, (b) volunteer work, (c) caregiving activities, (d) community involvement, (e) informal social interactions
Sabik, 2017, United States	Cross-sectional	123 Above 65 Female only	Survey	Assessed using a 20-item measure developed by Morgan et al. (1987).
Schladitz, 2022, Germany	Qualitative	18 Above 70 Both genders	Focus groups	
Seko, 2021, Japan	Cohort study	132 Aged 77.3 ± 5.3 Female only	Self-administered written questionnaire	Social activities: (a) neighborhood association activities, (b) older people’s club, (c) salon, (d) volunteer activities, (e) study sessions, (f) sports, (g) hobby/entertainment, (h) travel/visit
Siette, 2020, Australia	Cross-sectional	1,141 Above 75 Both genders	Extracted from Carelink+	Measured using the 15-item short form of the Australian Community Participation Questionnaire (ACPQ-SF15)
Sorensen, 2002, Denmark	Prospective cohort study	442 Aged 75–80 Both genders	Structured interview during a home visit	Operationalization of social participation by Avlund et al. was adopted.
Sousa, 2018, Brazil	Cross-sectional Prevalence	986 Aged 60–69 Both genders	Home interviews	Participation in four domains: (a) family circle, (b) sociocultural activities, (c) groups or associations, (d) religious practice
Takagi, 2013, Japan	Cohort study	2,728 Above 65 Both genders	Mailed postal survey	Assessed by asking whether the respondents participated in the 8 types of groups in their neighborhood
Thomas, 2011, United States	Panel study	1,642 Above 60–95 Both genders	Data from nationally representative panel study	Social engagement is a composite score indicating the frequency of involvement in five social activities: (a) talking on the phone with friends/family, (b) visiting with friends/family, (c) attending meetings/programs of groups or organizations, (d) attending religious services, (e) volunteering
Thompson, 2004, United States	Cohort study	135 Above 65 Male only	Questionnaire via interview	Measure of social participation – the social interaction men have with others who also provide them with specific types of emotional and instrumental social support.
Vega-Tinoco, 2022, Europe	Pseudo panel study	1,412 Above 50 (age stratified) Both genders	ESS	Respondents are asked which activities they have carried out in the last year in order to “improve things in [their country] or help prevent things from going wrong.” These questions include contact with politicians or governments, working in a political party or other type of organization or association, wearing a campaign badge or sticker, signing petitions, taking part in public demonstrations or boycotting products.
Wangliu, 2023, China	Cross-sectional	3,142 Above 65 Both genders	Survey	Assessed by asking “Do you perform the following activities regularly?.” The activities included outdoor activities, playing cards and mahjong, and organized social activities.
Ye, 2020, China	Longitudinal survey	8,117 Above 65 Both genders	Survey Chinese Longitudinal Heathy Longevity Survey (CLHLS)	Social engagement assessed by five dichotomous indicators: (a) marital status, (b) living arrangement, (c) availability of help when required, (d) participation in social activities
Yuta, 2018, Japan	Longitudinal study	3,380 Above 65 Both genders	Self-administered questionnaire	Using a scale from the Japan Gerontological Evaluation Study (JAGES)
Yuying, 2022, China	Cross-sectional	11,462 Above 60 Both genders	Survey China Longitudinal Aging Social Survey (CLASS)	The main questions asked in the questionnaire in relation to social participation include: “Can you take public transportation (such as bus) by yourself?,” “Can you shop by yourself?,” “Can you manage money yourself?,” “Are you currently engaged in income-generating work/ activity?,” “How many kinds of community activities did you take part in?”

In most quantitative studies, social participation was measured either dichotomously, i.e., engaged in social activity (yes/no), or with the frequency of social engagement (Table 3). Across the included studies, variability in the definitions, understandings, and measurements of social participation was observed. Only 19.0% (*n* = 8) of quantitative studies used a standardized measure of social participation such as the Older Adult Activity Inventory Questionnaire (49), Participation and Activity Limitation Survey (50), or 15-item short form of the Australian Community Participation Questionnaire (51). About one quarter (*n* = 13; 26.5%) of the quantitative studies were longitudinal, followed by 57.1% (*n* = 28) cross-sectional, and one prevalent (Table 3). For quantitative and mixed methods studies, the sample sizes ranged from 132 to 31,428, while it was from 18 to 89 for qualitative.

### 3.2 Quality of studies

Most studies had a clear aim, and the analyses and interpretations of findings were generally appropriate (Appendix C). The main methodological issue was potential measurement bias secondary to variations in social participation questionnaires used in quantitative studies, mostly relying on self-answered or assisted questionnaires.

### 3.3 Factors influencing social participation of older women and men

**Figure 2:**
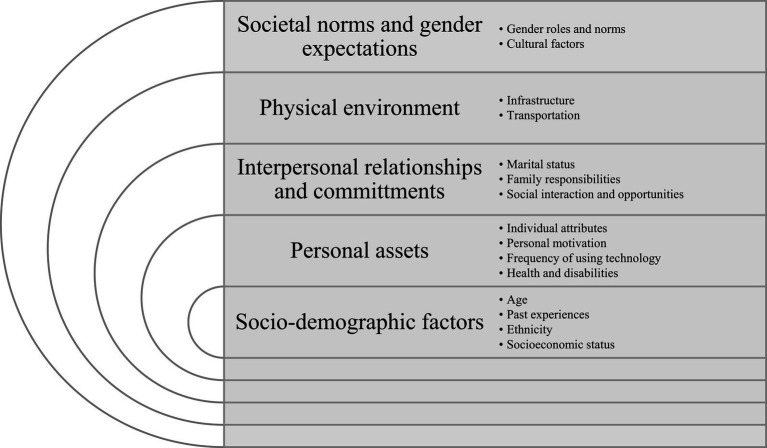
Summary of themes and descriptive factors influencing social participation among older men and women adults from socio-ecological perspective.

Individual and environmental factors influencing the social participation of older women and men were categorized in the following themes: (a) sociodemographic factors, (b) personal assets, (c) interpersonal relationships and commitments, (d) physical environment, and (e) societal norms and gender expectations (Figure 2). Overall, this review identified more evidence of factors influencing social participation among women than in men.

#### 3.3.1 Sociodemographic factors

At an individual level, sociodemographic factors, including age, past experiences, ethnicity, and socio-economic status shaped social participation among older women and men. Among women compared to men, advanced age and higher socio-economic status (SES) were found to have a greater influence, respectively negative and positive.

##### 3.3.1.1 Age

Social participation was found to decrease as both women and men get older (52, 53). Advanced age was reported as having a greater negative influence on women’s social participation (54, 55). Sex difference in social participation seemed to however disappear after 80 years old (56, 57). Participation in recreational activities was more frequent (OR = 0.42–0.45; *p* < 0.05) among both sexes from the 65–74 years old group compared to those 75 years and above (53) while receiving visitors at home increased during the ages from 75 to 80 (58).

##### 3.3.1.2 Past experiences

Earlier in their lives, women generally had more experience with primary relationships, e.g., with family and friends, and personal and intimate relations. Contrarily, men presented more secondary relationships, e.g., with people from the wider community, or participated in activities organized around narrower ranges of interests (60). Older women and men who engaged weekly in at least two of these social activities (visiting others, having visitors at home, participating in external social activities) earlier in their lives were more likely to have higher social participation in later years (58).

##### 3.3.1.3 Ethnicity

Ethnicity also had a differential effect on the social participation of older women and men (61–63). Among certain ethnic groups, i.e., Tamil and Sinhala, men had greater access to social activities than women (63). Social activities that older women and men engaged in also differed across ethnic groups. For example, compared to women, Chinese men participated less frequently in religious services but similarly in sports, while Malay and Indian men had higher participation in both activities (62).

##### 3.3.1.4 Socio-economic status

According to 14 studies, higher SES, specifically education level (64, 65), employment (53, 61), and financial status (52, 54, 66–73) positively influenced the ability to participate and access to social activities of older women and men. Generally, women had nevertheless lower SES and fewer resources than men (66). Thus, having higher financial support (74) and education level (54) greatly increased social participation among women more than men.

#### 3.3.2 Personal assets

The second theme provides valuable insights into the unique personal qualities and resources, comprising individual attributes, personal motivation, frequency of using technology, as well as health and disabilities, that influence social participation among older women and men. Particularly, the use of technology for health matters and having good health had a positive influence on the social participation of both women and men.

##### 3.3.2.1 Individual attributes

For both older women and men, individual attributes such as open-mindedness (70), satisfaction with life (75), orientation towards high social contribution (53), and a strong sense of community belonging (76) were found to be associated with greater social participation. The association between community belonging and social participation varied as a function of resilience, especially in men. Greater community belonging further enhanced social participation, especially among women (*p* = 0.03) and men (*p* < 0.01) with greater resilience (moderator effect).

##### 3.3.2.2 Personal motivation

Women and men reported similar motivations to engage in social activities such as coping with social isolation, reducing boredom and loneliness, keeping body and mind active, learning new things, and eating food (70, 77). Lack of interest and preference to be alone were reasons for not participating in social activities among both sexes (70). Participating in numerous interpersonal activities was found to predict increased Ikigai (sense of purpose), which was in turn found to be associated with greater motivation for new interactions with others among women (78). This association was not moderated by physical function (78). Men reported being too busy to participate more frequently than women did (79). While women focused on healthy eating (73), religious activities (70), and altruism through exchanging reciprocal aids, sharing values, and gaining emotional support (70, 80), men tend to emphasize maintaining an active lifestyle and engaging in meaningful activities (70, 73). Interestingly, experiencing positive emotions has been found to have a stronger influence on men’s social participation than on women (61).

##### 3.3.2.3 Frequency of using technology

Only one study examined the influence of Information Communication and Technology (ICT) usage and access on social participation (81). Having ICT access at any place and knowing how to use a computer increased social participation among women but not men. Using ICT for health matters increased family or friends visits among men and attendance for clubs, classes, and volunteering among women. ICT use for personal tasks was associated with decreased religious participation in both sexes.

##### 3.3.2.4 Health and disabilities

Good health was shown to have a positive influence on participation in social activities among both sexes (70, 72, 79, 82). Strong associations were observed between chronic diseases and social participation, through activity limitation (83). Older women and men who maintained high cognitive functioning reported greater participation in social activities (80, 84). Experiencing fewer depressive symptoms among women was found to be a ‘pre-requisite’ to participating in both collective and productive social activities (84). The results on the influence of good physical function on social participation according to sex were mixed. Functional disability and physical deterioration affected women’s social participation more in some studies (85, 86) while affecting men more according to others (61, 87).

#### 3.3.3 Interpersonal relationships and commitments

The third theme describes factors relating to interpersonal relationships and commitments, including marital status, family responsibilities, and social interactions and opportunities, which collectively influence social participation among older women and men. Having a larger social network generally increases the uptake of social activities while greater caregiving responsibilities reduce social participation among women and men. Specifically, marriage had a greater positive influence on men.

##### 3.3.3.1 Marital status

Older adults having a spouse reported greater abilities to engage in social activities independently compared to those without, but this positive influence was found to be lower in women than men (54). Married men tend to engage in more relationships (53, 88) and have more frequent and positive social participation (54, 68, 81). As they were expected to care for their partner’s needs, being married reduced women’s social participation (57), which can be explained by men’s reliance on their spouse for intimacy, emotional, instrumental, and caregiving support (67, 87). Conversely, widowed women were found to have a higher social participation. Despite the loss of their spouse, widows described positive aspects of being single, such as having personal freedom and not having to look after a partner (77). The importance of friendships during widowhood was salient; friends provided emotional support, encouragement to get outside, and fulfilled the desire to be with others. In addition, widowers typically reported having smaller friendship networks and, consequently, are at greater risk of social isolation than widows (89). Nonetheless and over time, widowhood positively influenced social participation more among men than in women (66). Widowers were more likely to participate in clubs or organizations, voluntary work, veteran groups, or conservation work compared to widows (66, 77, 89). Widowers were also found to be more likely to establish new intimate relationships, providing an important source of social contact and intimacy, which further emphasized men’s heavy reliance on support from women (77).

##### 3.3.3.2 Family responsibilities

Caregiving responsibilities, which are a competing priority among women compared to men, influence social participation. These responsibilities stemmed from the multifaceted demands placed upon women, encompassing responsibilities such as childcare, eldercare, and household management (80, 90). As the primary provider of family income, men were more involved in social activities (64). Meanwhile, married women staying at home with their children (65, 68) had restricted social participation due to their caregiving responsibilities (55, 65, 72, 76, 91–93). These caregiving duties often required a substantial time commitment and emotional investment, potentially limiting the availability and energy that women could allocate to social activities beyond their caregiving responsibilities. Once they were no longer caregivers, older women reported being able to form new social relationships and combat loneliness (57).

##### 3.3.3.3 Social interactions and opportunities

Social support is essential for the social participation of both women and men (68, 84), particularly emotional support (74). Men have been found to view social activities as opportunities to connect with others, expand their social networks, and foster new relationships (70). For both sexes, a larger network increases the probability of being introduced to various social groups, thus facilitating active social engagement (53). Women valued developing close interpersonal bonds, emphasizing the significance of cultivating strong relationships with others in their social networks (78). Notably, men who perceived themselves as having a greater number of reliable relationships tend to exhibit greater social participation (88). Positive social participation of men remains unaffected in the presence of conflicted relations (88). In this context, men displayed resilience to negative emotions and interactions and continued to actively participate in social activities.

Social network plays a significant role in influencing social participation, with both women and men acknowledging the importance of intergenerational contact for healthy aging (73). Men seemed to benefit mostly from socially active networks, while women relied on diverse networks (67). Women tend to engage more in social activities within the friends and community domains, whereas men primarily focused on immediate household interactions (53, 81, 82). Married women with limited social networks tend to exhibit restricted social participation (67).

By shaping an individual’s social interactions and networks, living arrangements also play a role in social participation. For instance, women living only with their spouses might experience a decrease in social activities, while those living with married children might witness a decline in childcare-centered activities (54, 65, 68). Unlike women, men living alone were less likely to be involved in any social group (53). The type of interactions and living arrangements thus influence social participation for both women and men.

#### 3.3.4 Physical environment

The fourth theme presents the crucial contributions of the physical environment in determining access to community spaces, namely infrastructure and transportation, which influence social participation among older women and men. Proximity to services and facilities as well as transportation were found to play an important role in facilitating women’s social participation.

##### 3.3.4.1 Infrastructure

Compared to men, women relied more on proximity services (94) and were more affected by living environments such as safety in the neighborhood, infrastructure development, and the presence of culturally restrictive norms or practices (54, 60, 65, 70) that might impact their ability to participate in social activities. Only one study reported greater perceived proximity to neighborhood resources enhanced social participation among women and men, but only in men with minor or no disability, i.e., not when having moderate to severe disabilities (95). Additionally, women were more likely to be satisfied with services from community organizations (60); while older men expressed the lack of specific social opportunities for themself after retirement: *“there are a lot more opportunities and things for women… men have other interests.”* (73). Although restricted social participation was observed among older adults from rural areas (73), men living in small towns were found to have greater social functioning than those in major metropolitan areas (52, 61). Moreover, men who have been long-time residents of their local community were more inclined to participate in formal social activities compared to other men with shorter duration of residence (53).

##### 3.3.4.2 Transportation

Not having a car or driving license, driving cessation and transportation problems restrict social participation among both women and men (73, 83, 94). The cessation or limitation in driving decreased mobility independence for older adults, as they had to rely on family members for assistance with transportation to events, appointments, and errands (77). The unreliability and inconvenience of taking public transport, as well as transportation challenges faced by individuals with disabilities, were reported by women and men (70). Compared to their younger counterparts, women older than 85 were more affected by transportation problems (71). Meanwhile, older men in urban centers reported transportation problems more often than rural ones (79).

#### 3.3.5 Societal norms and gender expectations

This last theme unveils how gender roles and norms, as well as culture, have an influence on the beliefs, societal views, and expectations of how women and men ought to behave and fulfill social roles which in turn influenced their social participation. Men were often viewed as the breadwinner while women as the caregiver which greatly influenced the frequency and type of social activities that they engaged in.

##### 3.3.5.1 Gender roles and norms

Traditional gender expectations in Asia assign men the role of the primary breadwinners, while women are often expected to be the primary caregivers within the household (86, 96, 97). This societal context influences men’s motivation and opportunities for social participation. Men who had the opportunity to tap into their abilities by taking leadership and organizational roles within friendship or alumni organizations (80, 98) might acquire greater motivation to engage in such activities (90). As they continued to maintain their roles as breadwinners, which enhanced their sense of belonging to society, and contributed to life satisfaction, paid work played a significant role in fostering men’s social participation (97). Men’s engagement in social activities was often linked to their primary role of providing family income (64), leading to high participation in social groups related to occupation, paid labor, and politics (56, 91, 97). Additionally, men who endorsed the traditional masculinity ideology seemed more satisfied with their social participation (88).

In contrast to men, women faced traditional gender role expectations that assign them additional responsibilities within the home, including housework and childcare (54). Compounded by the effects of the COVID-19 pandemic, these expectations increased the challenge for women to continue participating socially (93). In many cultures, women primarily took care of the family, while men undertook activities such as purchasing and social communication on behalf of the family (91). Women tend to engage in milder intensity social activities closer to the family and neighborhood nucleus (85), prioritizing family commitments and cultural activities as their primary means of being socially active (91, 93). Compared to men, women also presented a higher likelihood of participating in weekly religious group activities (55, 57, 97) and engaging in socializing with family or friends (70, 82). The social engagement of older women was often influenced by gender norms and societal expectations, which shaped the type of activities they participate in and the frequency of their engagement.

##### 3.3.5.2 Culture

Culture also influences social participation according to gender among older adults, as observed in various societies (55). In countries where patriarchal values are strong, such as Japan and China, men often sought meaning and identity through their valued roles in the workplace (99). Engaging in citizenship activities, which aligned with cultural notions of manhood, led to greater life satisfaction among men (98). While, in societies, such as Korea, where the family holds a central role, women assumed substantial caregiving and family responsibilities in late life, which, as mentioned, limited their opportunities for social participation (100). Additionally, cultural practices related to religion also shaped gender disparities in social activities (92). For instance, in Malay communities where Islam was practiced, differences between women and men were observed in rates of attendance at religious services due to religious expectations (62). These cultural variations provided insights into gender differences observed in the participation in specific social activities among older adults.

## 4 Discussion

This review offered a comprehensive socio-ecological perspective of how sex and gender play a role in social participation among older women and men. The findings identified how individual socio-demographic factors, personal assets, interpersonal relationships and commitments, access and adequacy of physical environment, societal norms and gender expectations influence the participation in social activities of older women and men. The consolidation of these evidence specific to each gender is unique and a key contribution to the existing literature. The findings of this review are salient in understanding how socially isolated older women and men can be approached to encourage and facilitate participation in social activities, mitigate loneliness, and cultivate habitual change in their social interactions. The factors identified in this review are discussed alongside practical implications for community practice and research.

### 4.1 Individual

The findings of the present study revealed that, compared to men, older women tend to have lower SES and financial resources to engage in social activities, which can lead to a higher risk of being under constrained circumstances and less inclined to participate socially. Advanced age also has a greater influence on women’s social involvement compared to men. This phenomenon can be attributed to the gender-health paradox, which posits that despite having a longer lifespan, women encounter a higher prevalence of chronic degenerative conditions that can limit their ability to participate in social activities (101). Additionally, because of their inclination toward intimate social connections, women are more susceptible to distress when someone they are emotionally close to, e.g., an immediate family member, close friend, or relative, undergoes stressful life events (102). Greater support might thus be necessary to assist older women experiencing depressive symptoms in discovering suitable social activities and increasing their motivation to participate purposefully. Consequently, participating in social activities might be more challenging for older women, as they may need to prioritize meeting the needs of other members within their social networks (103). Nevertheless, this does not imply that men consistently enjoy better health, higher SES, and greater social participation.

Sex differences in social participation were found to diminish after the age of 80, possibly as a result of reduced engagement in social activities and a shrinking social network. Pinto and Neri (33) noted that older men tend to withdraw from political and organizational activities, while women had a similar pattern for recreational, health-related, and informal social activities. As individuals age, their needs are found to become more diverse and complex, and their perspective of time and space shifts towards deeper matters such as existence and spirituality (104). Consequently, some social participation activities may have a lower priority for them in later life (105). This transcendent perspective may be influenced by an age-related health decline and increased risk of social isolation due to shrinking social networks (106, 107).

The present findings suggested that community care providers need to pay attention to older adults who are physically, socially, or economically disadvantaged, especially women, when planning and implementing community-based programs. While health or community care providers can refer these older adults to relevant social programs such as peer support groups and befriending, administrative processes of such referral services can be reviewed and simplified when possible, especially to alleviate inconveniences for individuals with fewer resources. Co-design and co-production of social and physical activities is an innovative and person-centered approach that can be used by community care providers to empower socially frail older adults, establish trust and relations, and develop sustainable solutions to engage them (108). Alternatively, regular surveys and focus groups can be conducted to gather feedback and better understand the evolving needs and preferences of specific sex, age, or other target groups of older adults. Additionally, leveraging ICT platforms such as videoconferencing (17) or customized innovative solutions such as virtual spaces (109) can provide a means to engage frail older women in the comfort of their homes. Since the association between social participation and health is bidirectional, active social engagement can also influence health, for example, by motivating a healthier lifestyle (110). More longitudinal studies considering the sex and gender perspective are also needed. Finally, policymakers should further play a critical role in allocating resources and funding to facilitate the implementation of effective social welfare programs that not only fulfill the basic needs of socially frail older adults but also cushion them from economic barriers and facilitate opportunities for social participation.

### 4.2 Interpersonal relationship

Interestingly, the present findings indicated that marriage offers men opportunities for companionship and social interactions with their spouse and others, resulting in increased social participation (111). For women, however, because they prioritize attending to the needs of their spouses and caregiving responsibilities, marriage tends to have the opposite effect. Consistent with the MacArthur Studies of Successful Aging, the findings of the present review supported that men primarily relied on their spouses for psychosocial support, whereas women are more supported by friends, relatives, and children (112). These findings are aligned with the socioemotional selectivity theory (113), which suggests that companionship becomes an increasingly essential motivator in later life. Given the differences in how marital status and social networks play a role in the socialization of older women and men, healthcare providers involved in social prescription and community care providers should consider the older adult’s familial interactions and commitments, and leverage these established connections to stimulate social participation. Other than organizing activities exclusively tailored for older adults, senior activity centers can collaborate with local community organizations and relevant agencies to plan purposeful community events or intergenerational activities that involve older adults’ existing social network, such as spouses, other family members, and friends. Older adults should also be given opportunities to volunteer with children and youth, as well as mentoring programs to facilitate meaningful relationships across different age groups and foster generativity and connection between generations (114). Future qualitative research should explore the perspectives and experiences of older couples to understand the contributory role of marriage and companionship in social participation. By exploring these elements, researchers will gain valuable insights and understanding about the dynamics of marital and familial relationships and their impact on older adults’ social engagement.

### 4.3 Community

Like past reviews (40, 115, 116), the present findings demonstrated that the availability, accessibility, and appeal of social spaces can significantly impact opportunities for social engagement among older adults. Yet, this review added that women were more likely to be limited by obstacles of the built environment, such as neighborhood safety, services provided in spatial proximity, and lack of accessibility to transport services, e.g., not possessing a driving license. The present findings suggested that increased outreach efforts, as well as ground up community initiatives, are needed to foster social participation among older women in neighborhoods with limited social amenities. On the other side, men were found to be less satisfied with social opportunities and lacked appeal towards these activities and programs available in community organizations. Firstly, this might be due to perceived gender norms toward certain organized social activities, e.g., flower arrangement and cooking that are perceived as feminized or domestic activities. Secondly, perceived gender norms regarding men upholding stereotypical masculinity of being hyper-independent, strong, and stoic individuals and women easily affiliating with close interpersonal relationships in social settings might make the participation in organized social activities more socially appealing and appropriate for women. Community organizations serving older adults need to rethink and provide a combination of sex-specific and neutral activities that interest both older women and men. The present findings also reiterated the importance of considering proximity as well as ease of access and travel to community spaces during land use planning at the municipality level. This consideration requires dialogue and multisectoral collaboration among public health advocates, gerontologists, policymakers, and land use planners to implement experimental and structural interventions within neighborhoods to create an age-friendly and sustainable environment (115).

### 4.4 Societal factors

The present findings accentuated the sociocultural perceptions that femininity and masculinity affect the type of activity in which older adults participate. The social role theory posits cultural norms and expectations regarding gender roles that shape individuals’ behavior and adherence to these societal beliefs (117). Men’s social engagement often revolves around work and tends to decrease after retirement, while women’s social engagement tends to be more centered around family and continues even after retirement (118). This can be attributed to men associating their self-identity with work and occupational status, while women priorities relationships as integral to their identity (117). Often, men are perceived as the family breadwinners and women as caregivers (119). Such gender norms thus prevent most men from assuming equal caregiving responsibilities at home. As men retire, formal social participation becomes crucial in filling the vacated roles and preserving the continuity of social relationships (120). For women who have been homemakers, religious activities can offer non-material support and a sense of belonging (120). Given that men often derive their self-identity and contributory role from their occupations, having to replace, recreate, or maintain sources of meaning acquired from work life is crucial while transiting to and during retirement (121). The present findings suggested that healthcare providers involved in social prescription and community care providers attending to older men should guide conversations in exploring activities that are perceived personally valuable and acknowledged by others, as well as those that seek to reestablish a sense of being part of a social group (121). By facilitating open dialogues with both older women and men on their preferences, aspirations, personal strengths, and past experiences of social participation, healthcare providers and community care providers can also enhance their self-awareness, receptivity, and empathy, thereby fostering a more sex and gender-inclusive and accepting community. From a broader perspective, with increasing economic participation among women and dual-career households, perceptions towards sociocultural norms and roles of women and men among future cohorts of older adults may represent more openness and gender neutrality. Nonetheless, more longitudinal studies are needed to verify if differences in social participation are attributable to gender or individual differences, especially among future cohorts of older adults.

Finally, this review revealed a need to identify more factors that influence men’s social participation than women. Although the specific nature of these findings — limited evidence on factors influencing social participation among older men remains to be confirmed, it is important to promote and facilitate men’s engagement in social activities, as they generally present smaller social networks, and consequently being more at risk of social isolation (122–124). To ensure that community programs are accessible and appealing to all, it is crucial for healthcare providers involved in social prescription and community care providers to acknowledge and address men’s unique needs and preferences.

### 4.5 Strengths and limitations

This review’s main strength is the integration of diverse study designs which comprehensively addresses the review question. As such, the findings can inform relevant community care providers regarding the development and implementation of social programs and activities from a gender perspective. This review also included studies that were conducted across countries with differing cultural and social contexts, though the majority of them were from Asia. Nonetheless, cultural or cross-geographic differences pertaining to the influencing factors of social participation among older women and men were not observed in this review. This might be attributed to the inherent heterogeneity in cultural, social, and healthcare contexts between regions, such as European and Asian countries. Alternatively, our choice of data analysis approach involves integrating data from the included studies which amalgamates findings across diverse geographical regions and cultural contexts, and this might have limited direct cultural or cross-geographical comparisons.

Due to unavailable literature on gender, one key limitation of this review is the lack of inclusion of older adults with alternate sexual orientation and gender identity, such as the lesbian, gay, bisexual, and transgender community. None of the studies make the distinction between sex and gender, making the understanding blurred. Also, it was more difficult to compare qualitative results by sex as differences relied on a small sample and might be due to personal preferences. Studies not available in full texts or published in non-English languages were excluded, so potential studies may have been missed (125). Furthermore, variability in how social participation was measured across studies had influenced the result of the current review; Only 19% (*n* = 8) of studies used standardized measures of social participation. Although some studies combined standardized measures and unvalidated measures, most studies developed their method of measuring participation based on a combination of frequency of attendance, social network size, or descriptive interviews. Restricted use of social participation definitions and associated measures may limit the generalizability of the findings of the present study and challenge the development of clear guidelines for policymakers, practitioners, or researchers.

## 5 Conclusion

This systematic review synthesized the findings on the available evidence of factors influencing the social participation of older women and men using the socio-ecological health lens. The findings highlighted the importance of considering the heterogeneous needs, preferences, and inequalities faced by older women and men, as well as recognizing societal norms and expectations surrounding gender when planning and implementing programs and creating adequate and accessible social spaces. Special attention is needed among community care providers and healthcare professionals to co-design, implement, and prescribe a combination of sex and gender-specific and neutral activities that interest both older women and men. Intersectoral collaborative efforts, including public health advocates, gerontologists, policymakers, and land use planners are needed to unify efforts to foster social inclusivity by creating an age-friendly and sustainable physical and social environment. More longitudinal studies are required to better understand social participation trajectories from a sex and gender perspective and identify factors influencing it.

## Data availability statement

The original contributions presented in the study are included in the article/Supplementary material, further inquiries can be directed to the corresponding author/s.

## Author contributions

OCH: Conceptualization, Data curation, Formal analysis, Methodology, Project administration, Validation, Visualization, Writing – original draft, Writing – review & editing. PBL: Data curation, Validation, Writing – review & editing. ML: Supervision, Writing – review & editing. GRT: Formal analysis, Validation, Writing – review & editing. BS: Conceptualization, Formal analysis, Methodology, Project administration, Resources, Supervision, Validation, Visualization, Writing – original draft, Writing – review & editing.
