# Supplementary material for: Sex and gender differences in social participation among community-dwelling older adults: a systematic review
Source: Front Public Health. 2024 Apr 12;12:1335692. doi: 10.3389/fpubh.2024.1335692 (PMC11046488; doi:10.3389/fpubh.2024.1335692)
Supplement: Supplementary file 1 [file Data_Sheet_1.docx]

Appendix A: Preferred Reporting Items for Systematic Reviews and Meta Analyses (PRISMA) guideline

| **Section and Topic** | **Item #** | **Checklist item** | **Location where item is reported** |
| --- | --- | --- | --- |
| **TITLE** | | |  |
| Title | 1 | Identify the report as a systematic review. | 1 |
| **ABSTRACT** | | |  |
| Abstract | 2 | See the PRISMA 2020 for Abstracts checklist. | 1 |
| **INTRODUCTION** | | |  |
| Rationale | 3 | Describe the rationale for the review in the context of existing knowledge. | 2 |
| Objectives | 4 | Provide an explicit statement of the objective(s) or question(s) the review addresses. | 2 |
| **METHODS** | | |  |
| Eligibility criteria | 5 | Specify the inclusion and exclusion criteria for the review and how studies were grouped for the syntheses. | 2-3 |
| Information sources | 6 | Specify all databases, registers, websites, organisations, reference lists and other sources searched or consulted to identify studies. Specify the date when each source was last searched or consulted. | 3 |
| Search strategy | 7 | Present the full search strategies for all databases, registers and websites, including any filters and limits used. | Appendix B |
| Selection process | 8 | Specify the methods used to decide whether a study met the inclusion criteria of the review, including how many reviewers screened each record and each report retrieved, whether they worked independently, and if applicable, details of automation tools used in the process. | 3 |
| Data collection process | 9 | Specify the methods used to collect data from reports, including how many reviewers collected data from each report, whether they worked independently, any processes for obtaining or confirming data from study investigators, and if applicable, details of automation tools used in the process. | 3-4 |
| Data items | 10a | List and define all outcomes for which data were sought. Specify whether all results that were compatible with each outcome domain in each study were sought (e.g. for all measures, time points, analyses), and if not, the methods used to decide which results to collect. | 3-4 |
|  | 10b | List and define all other variables for which data were sought (e.g. participant and intervention characteristics, funding sources). Describe any assumptions made about any missing or unclear information. | 3-4 |
| Study risk of bias assessment | 11 | Specify the methods used to assess risk of bias in the included studies, including details of the tool(s) used, how many reviewers assessed each study and whether they worked independently, and if applicable, details of automation tools used in the process. | 4 |
| Effect measures | 12 | Specify for each outcome the effect measure(s) (e.g. risk ratio, mean difference) used in the synthesis or presentation of results. | N.A. |
| Synthesis methods | 13a | Describe the processes used to decide which studies were eligible for each synthesis (e.g. tabulating the study intervention characteristics and comparing against the planned groups for each synthesis (item #5)). | 4 |
|  | 13b | Describe any methods required to prepare the data for presentation or synthesis, such as handling of missing summary statistics, or data conversions. | 4 |
|  | 13c | Describe any methods used to tabulate or visually display results of individual studies and syntheses. | 4 |
|  | 13d | Describe any methods used to synthesize results and provide a rationale for the choice(s). If meta-analysis was performed, describe the model(s), method(s) to identify the presence and extent of statistical heterogeneity, and software package(s) used. | 4 |
|  | 13e | Describe any methods used to explore possible causes of heterogeneity among study results (e.g. subgroup analysis, meta-regression). | N.A. |
|  | 13f | Describe any sensitivity analyses conducted to assess robustness of the synthesized results. | N.A. |
| Reporting bias assessment | 14 | Describe any methods used to assess risk of bias due to missing results in a synthesis (arising from reporting biases). | N.A. |
| Certainty assessment | 15 | Describe any methods used to assess certainty (or confidence) in the body of evidence for an outcome. | N.A. |
| **RESULTS** | | |  |
| Study selection | 16a | Describe the results of the search and selection process, from the number of records identified in the search to the number of studies included in the review, ideally using a flow diagram. | Figure 1 |
|  | 16b | Cite studies that might appear to meet the inclusion criteria, but which were excluded, and explain why they were excluded. | N.A. |
| Study characteristics | 17 | Cite each included study and present its characteristics. | Table 3 |
| Risk of bias in studies | 18 | Present assessments of risk of bias for each included study. | Appendix C |
| Results of individual studies | 19 | For all outcomes, present, for each study: (a) summary statistics for each group (where appropriate) and (b) an effect estimate and its precision (e.g. confidence/credible interval), ideally using structured tables or plots. | N.A. |
| Results of syntheses | 20a | For each synthesis, briefly summarise the characteristics and risk of bias among contributing studies. | Appendix C, 4 |
|  | 20b | Present results of all statistical syntheses conducted. If meta-analysis was done, present for each the summary estimate and its precision (e.g. confidence/credible interval) and measures of statistical heterogeneity. If comparing groups, describe the direction of the effect. | N.A. |
|  | 20c | Present results of all investigations of possible causes of heterogeneity among study results. | N.A. |
|  | 20d | Present results of all sensitivity analyses conducted to assess the robustness of the synthesized results. | N.A. |
| Reporting biases | 21 | Present assessments of risk of bias due to missing results (arising from reporting biases) for each synthesis assessed. | N.A. |
| Certainty of evidence | 22 | Present assessments of certainty (or confidence) in the body of evidence for each outcome assessed. | N.A. |
| **DISCUSSION** | | |  |
| Discussion | 23a | Provide a general interpretation of the results in the context of other evidence. | 12-14, Figure 2 |
|  | 23b | Discuss any limitations of the evidence included in the review. | 14-15 |
|  | 23c | Discuss any limitations of the review processes used. | 14-15 |
|  | 23d | Discuss implications of the results for practice, policy, and future research. | 13-15 |
| **OTHER INFORMATION** | | |  |
| Registration and protocol | 24a | Provide registration information for the review, including register name and registration number, or state that the review was not registered. | 2 |
|  | 24b | Indicate where the review protocol can be accessed, or state that a protocol was not prepared. | 2 |
|  | 24c | Describe and explain any amendments to information provided at registration or in the protocol. | N.A. |
| Support | 25 | Describe sources of financial or non-financial support for the review, and the role of the funders or sponsors in the review. | 15 |
| Competing interests | 26 | Declare any competing interests of review authors. | 15 |
| Availability of data, code and other materials | 27 | Report which of the following are publicly available and where they can be found: template data collection forms; data extracted from included studies; data used for all analyses; analytic code; any other materials used in the review. | 15 |

Appendix B: Full search strategy across the databases

|  | | **Concept 1 /Population/Problem** | **Concept2**  **/Intervention/Exposure/Interest** | **Additional concept (if any) /comparison/outcome** |
| --- | --- | --- | --- | --- |
| **Key concepts**  *Identify the key concepts based on your research topic.* | | Community-dwelling older adults | Social participation | Gender difference |
| **Free text terms / natural language terms**  (synonyms, UK/US terminology, medical/laymen’s terms, acronyms/abbreviations, drug brands, more narrow search terms)  *List down your keywords for each concept.* | | “Old* adult*” OR  “Old* people” OR  “Old age” OR  “Advanced age” OR  “Aged”  “aged 60” OR “60+” OR  “aged people” OR  “Aged person” OR  “aged subject” OR  “ag?ing” OR  “elder*”OR  “elderly people” OR  “elderly person” OR  “elderly subject” OR  “Elderly adult*” OR  “senior*” OR  “senior citizen” OR  “Senium” OR  “Geriatric*” | “social participation” OR  “social engagement” OR  “social involvement” OR  “social activit*” OR  “social inclusion” OR  “social connect*” OR  “Community participation” OR  “Community involvement” OR  “Community engagement” OR  “community group*” OR  “Civic participation” OR  “Social integration” OR  “Social contact*” OR  “Social interaction*” OR  “social network*” OR  “productive activit*” OR  “leisure activit*” OR “leisure” OR “leisure time” OR  “social capital” | “Gender differen*” OR  “Sex* differen*” OR  “Gender role*” OR  “Male vs female” OR  “Female vs male” OR  “Men vs women” OR  “Women vs men” OR  “Man vs woman” OR  “Woman vs man” OR  “men and women” OR  “Women and men” OR  “Man and woman” OR  “Woman and man” OR  “Femininity and masculinity” OR  “Masculinity and femininity” |
| **Medline (PubMed)* / Cochrane Library)** | **Controlled vocabulary** **terms**  MeSH terms | Aged[Mesh] | social participation[Mesh] OR  social capital[Mesh] OR  leisure activities[Mesh] | Sex characteristics[Mesh] OR  Gender role[Mesh] |
|  | **Keyword statement** | “old* adult*”[Title/Abstract] OR  “elder*” [Title/Abstract] OR  “aged”[Title/Abstract] OR  “aging”[Title/Abstract] OR  “ageing” [Title/Abstract] OR  “geriatric*”[Title/Abstract] OR  “senior*”[Title/Abstract] OR  “older people”[Title/Abstract] OR  “old people”[Title/Abstract] OR  “Old age”[Title/Abstract] OR  “Advanced age”[Title/Abstract] OR  “aged 60”[Title/Abstract] OR “60+”[Title/Abstract] OR  “aged people”[Title/Abstract] OR  “Aged person”[Title/Abstract] OR  “aged subject”[Title/Abstract] OR  “elderly people”[Title/Abstract] OR  “elderly person”[Title/Abstract] OR  “elderly subject”[Title/Abstract] OR  “Elderly adult*”[Title/Abstract] OR  “senior citizen”[Title/Abstract] OR  “Senium”[Title/Abstract] | “social participation”[Title/Abstract] OR  “social engagement”[Title/Abstract] OR  “social involvement”[Title/Abstract] OR  “social activit*”[Title/Abstract] OR  “social inclusion”[Title/Abstract] OR  “social connect*”[Title/Abstract] OR  “social network*” [Title/Abstract] OR  “productive activit*”[Title/Abstract] OR  “leisure activit*”[Title/Abstract] OR “leisure”[Title/Abstract] OR “leisure time”[Title/Abstract]  “social capital”[Title/Abstract] OR  “community group*”[Title/Abstract] OR  “Community participation”[Title/Abstract] OR  “Community involvement” [Title/Abstract] OR  “Community engagement” [Title/Abstract] OR  “Civic participation”[Title/Abstract] OR  “Social integration”[Title/Abstract] OR  “Social contact*”[Title/Abstract] OR  “Social interaction”[Title/Abstract] | “gender differen*”[Title/Abstract] OR “sex* differen*”[Title/Abstract] OR “gender role*”[Title/Abstract] OR “male vs female”[Title/Abstract] OR “female vs male”[Title/Abstract]  “men vs women”[Title/Abstract] OR “Women vs men”[Title/Abstract] OR  “Man vs woman”[Title/Abstract] OR  “Woman vs man”[Title/Abstract] OR  “men and women”[Title/Abstract] OR  “Women and men”[Title/Abstract] OR  “Man and woman”[Title/Abstract] OR  “Woman and man”[Title/Abstract] OR  “Femininity and masculinity”[Title/Abstract] OR  “Masculinity and femininity”[Title/Abstract] |
| **EMBASE.com** | **Controlled vocabulary** **terms**  Emtree terms | ‘Aged’/exp OR  ‘Aging’/exp OR  ‘Geriatrics’/exp OR  ‘Older people’/exp OR  ‘Older adult’/exp OR  ‘Older adults’/exp | ‘Social participation’/exp OR  ‘Social capital’/exp OR  ‘Social engagement’/exp OR  ‘Social inclusion’/exp OR  ‘Social connectedness’/exp OR  ‘Social network’/exp OR  ‘Leisure’/exp OR  ‘Community participation’/exp OR  ‘Community engagement’/exp OR  ‘Social interaction’/exp | ‘Sexual characteristics’/exp OR  ‘Gender differences’/exp OR  ‘Sex difference’/exp OR  ‘Sex role’/exp |
|  | **Keyword statement** | ‘old* adult*’:ti,ab OR ‘old* people’:ti,ab OR ‘old age’:ti,ab OR ‘advanced age’:ti,ab OR ‘aged 60’:ti,ab OR ‘60+’:ti,ab OR ‘aged people’:ti,ab OR ‘aged person’:ti,ab OR ‘aged subject’:ti,ab OR ‘ag?ing’:ti,ab OR ‘elder*’:ti,ab OR ‘elderly people’:ti,ab OR ‘elderly person’:ti,ab OR ‘elderly subject’:ti,ab OR ‘elderly adult*’:ti,ab OR ‘senior*’:ti,ab OR ‘senior citizen’:ti,ab OR ‘senium’:ti,ab OR ‘geriatric*’:ti,ab | ‘social participation’:ti,ab OR ‘social engagement’:ti,ab OR ‘social involvement’:ti,ab OR ‘social activit*’:ti,ab OR ‘social inclusion’:ti,ab OR ‘social connect*’:ti,ab OR ‘social network*’:ti,ab OR ‘productive activit*’:ti,ab OR ‘leisure’:ti,ab OR ‘leisure activit*’:ti,ab OR ‘leisure time’:ti,ab OR ‘social capital’:ti,ab OR ‘community group*’:ti,ab OR ‘community participation’:ti,ab OR ‘community engagement’:ti,ab OR ‘community involvement’:ti,ab OR ‘civic participation’:ti,ab OR ‘social integration’:ti,ab OR ‘social contact*’:ti,ab OR ‘social interaction*’:ti,ab | ‘sexual characteristic*’:ti,ab OR  ‘sex characteristic*’:ti,ab OR  ‘differen*, sex’:ti,ab OR  "gender differen*":ti,ab OR "sex differen*":ti,ab OR  ‘sexual differen*’:ti,ab OR  ‘Gender role*’:ti,ab OR  ‘male vs female’:ti,ab OR ‘female vs male’:ti,ab OR  ‘men vs women’ :ti,ab OR ‘women vs men’:ti,ab OR ‘man vs woman’:ti,ab OR ‘woman vs man’:ti,ab OR ‘men and women’:ti,ab OR ‘women and men’:ti,ab OR ‘man and woman’:ti,ab OR ‘woman and man’:ti,ab OR ‘femininity and masculinity’:ti,ab OR ‘masculinity and femininity’:ti,ab |
| **CINAHL Complete* (EBSCO)** | **Controlled vocabulary** **terms**  CINAHL Subject Headings | MH “aged” | MH “social participation” or  MH “social capital” or  MH “social networks” | MH “gender role” |
|  | **Keyword statement** | AB (aged or elder* or elderly people Or elderly person or elderly subject or elderly adult* or old* adult* or geriatric* or aging or ageing or senior* or senior citizen or senium or old* people or old age or advanced age or aged people or aged person or aged subject or ageing or aging or aged 60 or 60+) | AB (social participation or social engagement or social activit* or social involvement or social capital or social network* or community group* or social inclusion OR social connect* OR productive activit* or leisure activit* or leisure time or leisure or Community participation or community involvement or Community engagement or Civic participation or Social integration or Social contact* or Social interaction*) | AB (gender differen* or sex differen* or sexual differen* or male vs female Or female vs male or men vs women OR women vs men or man vs woman or woman vs man or gender role* or men women or women men or man woman or woman man or femininity masculinity or masculinity femininity) |
| **PsycINFO***  **(Ovid)** | **Controlled vocabulary** **terms**  Thesaurus of Psychological Index Terms® | (Aging OR aging in place OR healthy aging OR aged OR geriatrics OR gerontology OR life changes).sh | (Social influences OR social networks OR social capital OR social connectedness OR social interaction OR social support OR social behaviour).sh | (human sex differences OR sex OR group differences OR sex role attitudes OR attitudes OR sex roles OR stereotyped attitudes).sh |
|  | **Keyword statement** | (Old* adult* OR old* people OR old age OR advanced age OR elder* OR aged OR aged 60 OR 60+ OR aged people OR aged person OR aged subject OR elderly people OR elderly person OR elderly subject OR elderly adult* OR senior citizen OR senium OR ag?ing OR geriatric* OR senior*).ab,ti | (social participation or social engagement or social involvement or social activit* or social inclusion or social connect* or social network* or productive activit* or leisure activit* or leisure or leisure time or social capital or community group* or Community participation or Community involvement or Community engagement or Civic participation or Social integration or Social contact* or Social interaction*).ab,ti | (Gender differen* or sexual differen* or Sex differen* or Gender role* or Male vs female or female vs male or Men vs women or women vs men or man vs woman or woman vs man or men women or women men or man woman or woman man or femininity masculinity or masculinity femininity).ab,ti |
| **Social Science Database (ProQuest)** | **Controlled vocabulary terms**  Thesaurus/  Subject Headings | subject(“aging” OR “older people” OR “geriatric”) | subject(“social participation” OR “social networks” OR “connectedness”) | subject(“gender differences” OR “sex differences” OR “sex roles”) |
|  | **Keyword statement** | noft(“Old* adult*” OR “Old* people” OR “old age” OR “advanced age” OR “aged” OR “aged 60” OR “60+” OR “aged people” OR “Aged person” OR “aged subject” OR “aging” OR “elder*” OR “elderly people” OR “elderly person” OR “elderly subject” OR “elderly adult*” OR “senior*” OR “senior citizen” OR “Senium” OR “Geriatric*”) | noft(“social participation” OR “social engagement” OR “social involvement” OR “social activit*” OR “social inclusion” OR “social connect*” OR “Community participation” OR “Community involvement” OR “Community engagement” OR “community group*” OR “Civic participation” OR “Social integration” OR “Social contact*” OR “Social interaction*” OR “social network*” OR “productive activit*” OR “leisure activit*” OR “leisure” OR “leisure time” OR “social capital”) | noft(“Gender differen*” OR “Sex* differen*” OR “Gender role*” OR “Male vs female” OR “Female vs male” OR “Men vs women” OR “Women vs men” OR “man vs woman” OR “woman vs man” OR “men and women” OR “women and men” OR “man and woman” OR “woman and man” OR “femininity and masculinity” OR “masculinity and femininity”) |
| **Scopus** | **Keyword statement** | TITLE-ABS-KEY  “Old* adult*” OR  “Old* people” OR  “Old age” OR  “Advanced age” OR  “Aged” OR  “aged 60” OR “60+” OR  “aged people” OR  “Aged person” OR  “aged subject” OR  “ag?ing” OR  “elder*”OR  “elderly people” OR  “elderly person” OR  “elderly subject” OR  “Elderly adult*” OR  “senior*” OR  “senior citizen” OR  “Senium” OR  “Geriatric*” | TITLE-ABS-KEY  “social participation” OR  “social engagement” OR  “social involvement” OR  “social activit*” OR  “social inclusion” OR  “social connect*” OR  “social network*” OR  “productive activit*” OR  “leisure activit*” OR “leisure” OR “leisure time” OR  “social capital” OR  “community group*” OR “Community participation” OR “Community involvement” OR “Community engagement” OR “Civic participation” OR “Social integration” OR “Social contact*” OR “Social interaction*” | TITLE-ABS-KEY  “gender differen*” OR “sex differen*” OR “Sexual differen*” OR  “Gender role*” OR “male vs female” OR “female vs male” OR “men vs women” OR “women vs men” OR “man vs woman” OR “woman vs man” OR “men and women” OR “women and men” OR “man and woman” OR “woman and man” OR “femininity and masculinity” OR “masculinity and femininity” |
| **Web of Science**  **Core collection** | **Keyword statement** | **TS=(“**Old* adult*” OR  “Old* people” OR  “Old age” OR  “Advanced age” OR  “Aged” OR  “aged 60” OR “60+” OR  “aged people” OR  “Aged person” OR  “aged subject” OR  “ag?ing” OR  “elder*”OR  “elderly people” OR  “elderly person” OR  “elderly subject” OR  “Elderly adult*” OR  “senior*” OR  “senior citizen” OR  “Senium” OR  “Geriatric*) | **TS=(“**social participation” OR  “social engagement” OR  “social involvement” OR  “social activit*” OR  “social inclusion” OR  “social connect*” OR  “social network*” OR  “productive activit*” OR  “leisure activit*” OR “leisure” OR “leisure time” OR  “social capital” OR  “community group*” OR “Community participation” OR “Community involvement” OR “Community engagement” OR “Civic participation” OR “Social integration” OR “Social contact*” OR “Social interaction*”) | **TS=(“gender differen*” OR “sex differen*” OR “Sexual differen*” OR “Gender role*” OR “male vs female” OR “female vs male” OR “men vs women” OR “women vs men” OR “man vs woman” OR “woman vs man” OR “men and women” OR “women and men” OR “man and woman” OR “woman and man” OR “femininity and masculinity” OR “masculinity and femininity”)** |
| **ASSIA (Proquest)** | **Controlled vocabulary** **terms** | Subject(“Older people” OR “aging” OR “elderly people” or “aged”) | Subject(“Social participation” or “social networks” or “social interaction” or “social interactions” or “social integration” or “Leisure activities”) | Subject(“Gender differences” or “gender” or “sex differences” or “gender aspects” or “sex roles” or “sex factors”) |
|  | **Keyword statement** | noft(“Old* adult*” OR “Old* people” OR “old age” OR “advanced age” OR “aged” OR “aged 60” OR “60+” OR “aged people” OR “Aged person” OR “aged subject” OR “ag?ing” OR “elder*” OR “elderly people” OR “elderly person” OR “elderly subject” OR “elderly adult*” OR “senior*” OR “senior citizen” OR “Senium” OR “Geriatric*”) | noft(“social participation” OR “social engagement” OR “social involvement” OR “social activit*” OR “social inclusion” OR “social connect*” OR “Community participation” OR “Community involvement” OR “Community engagement” OR “community group*” OR “Civic participation” OR “Social integration” OR “Social contact*” OR “Social interaction*” OR “social network*” OR “productive activit*” OR “leisure activit*” OR “leisure” OR “leisure time” OR “social capital”) | noft(“Gender differen*” OR “Sex* differen*” OR “Gender role*” OR “Male vs female” OR “Female vs male” OR “Men vs women” OR “Women vs men” OR “man vs woman” OR “woman vs man” OR “men and women” OR “women and men” OR “man and woman” OR “woman and man” OR “femininity and masculinity” OR “masculinity and femininity”) |
| **Proquest Dissertations and Theses Global** | **Controlled vocabulary** **terms** | Subject(“Gerontology” OR “aging” OR “older people”) | Subject(“Sociology” OR “social work” OR “Social research” OR “Social structure” OR “Social support”) | Subject(“Gender studies”) |
|  | **Keyword statement** | noft(“Old* adult*” OR “Old* people” OR “old age” OR “advanced age” OR “aged” OR “aged 60” OR “60+” OR “aged people” OR “Aged person” OR “aged subject” OR “ag?ing” OR “elder*” OR “elderly people” OR “elderly person” OR “elderly subject” OR “elderly adult*” OR “senior*” OR “senior citizen” OR “Senium” OR “Geriatric*”) | noft(“social participation” OR “social engagement” OR “social involvement” OR “social activit*” OR “social inclusion” OR “social connect*” OR “Community participation” OR “Community involvement” OR “Community engagement” OR “community group*” OR “Civic participation” OR “Social integration” OR “Social contact*” OR “Social interaction*” OR “social network*” OR “productive activit*” OR “leisure activit*” OR “leisure” OR “leisure time” OR “social capital”) | noft(“Gender differen*” OR “Sex* differen*” OR “Gender role*” OR “Male vs female” OR “Female vs male” OR “Men vs women” OR “Women vs men” OR “man vs woman” OR “woman vs man” OR “men and women” OR “women and men” OR “man and woman” OR “woman and man” OR “femininity and masculinity” OR “masculinity and femininity”) |

Appendix C: Quality appraisal of included studies

Figure C1: Critical appraisal of cross-sectional studies (McGuinness & Higgins, 2020)

**
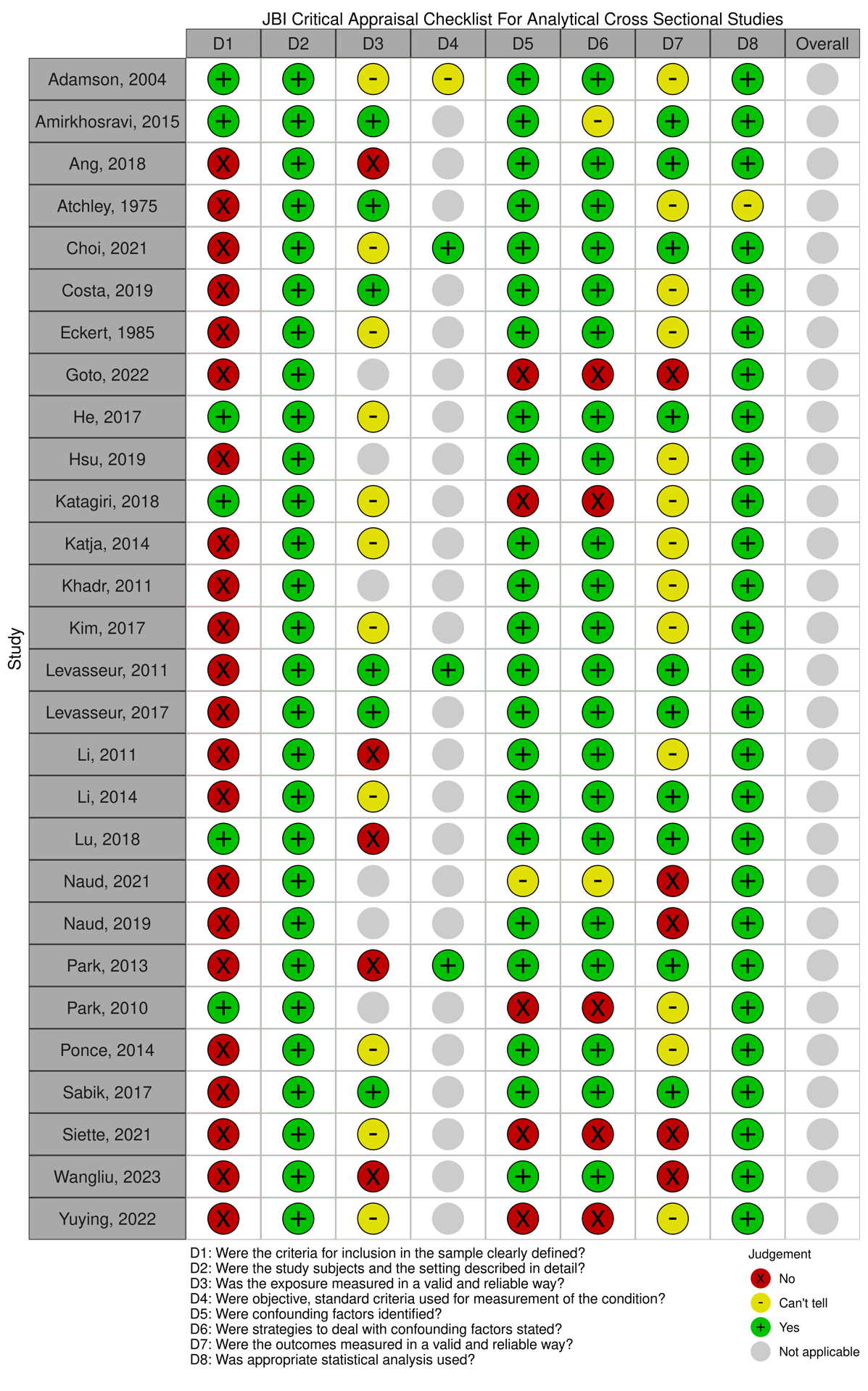
**

Figure C2: Critical appraisal of cohort studies (McGuinness & Higgins, 2020)


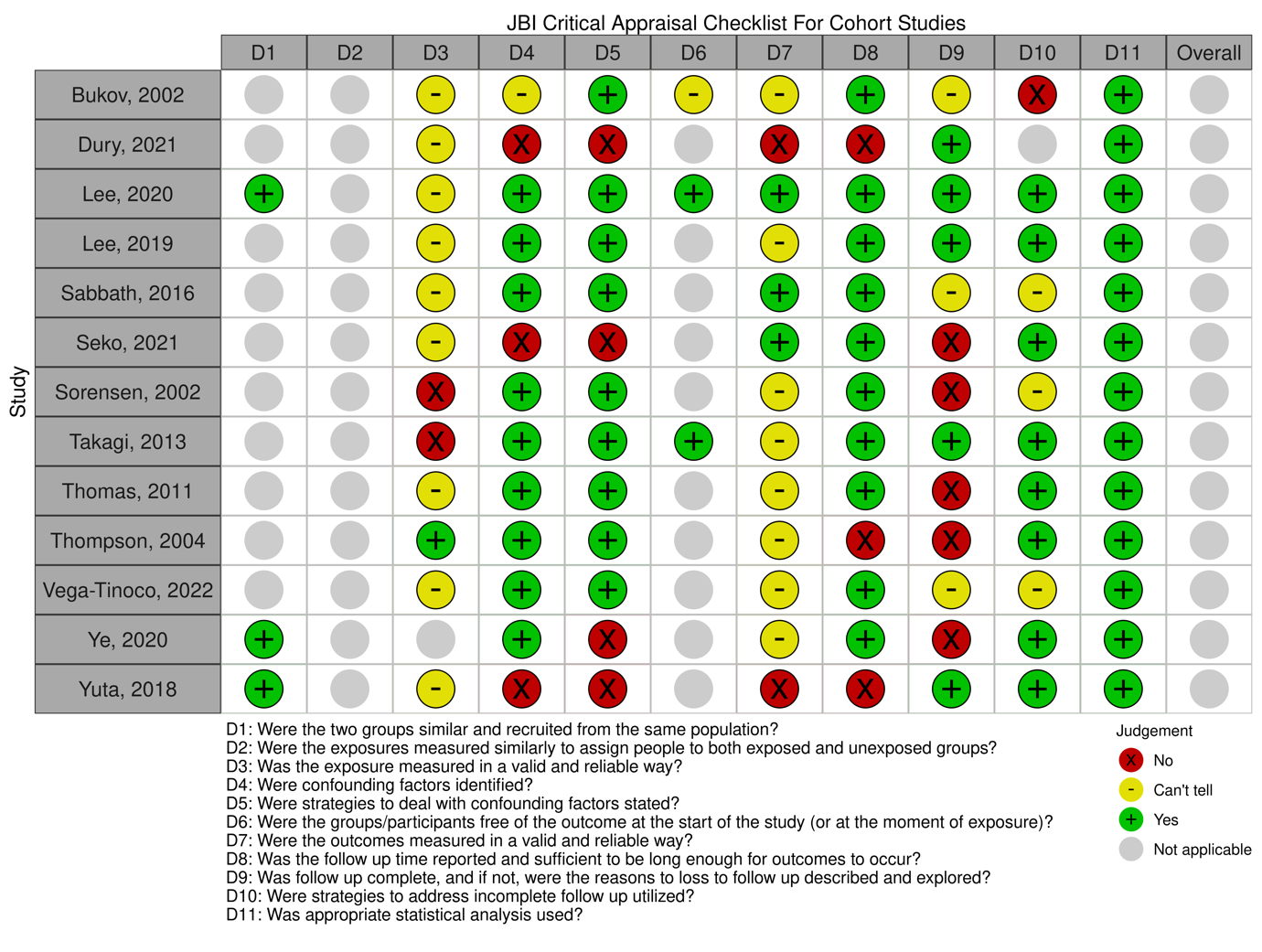


Figure C3: Critical appraisal of prevalent studies (McGuinness & Higgins, 2020)


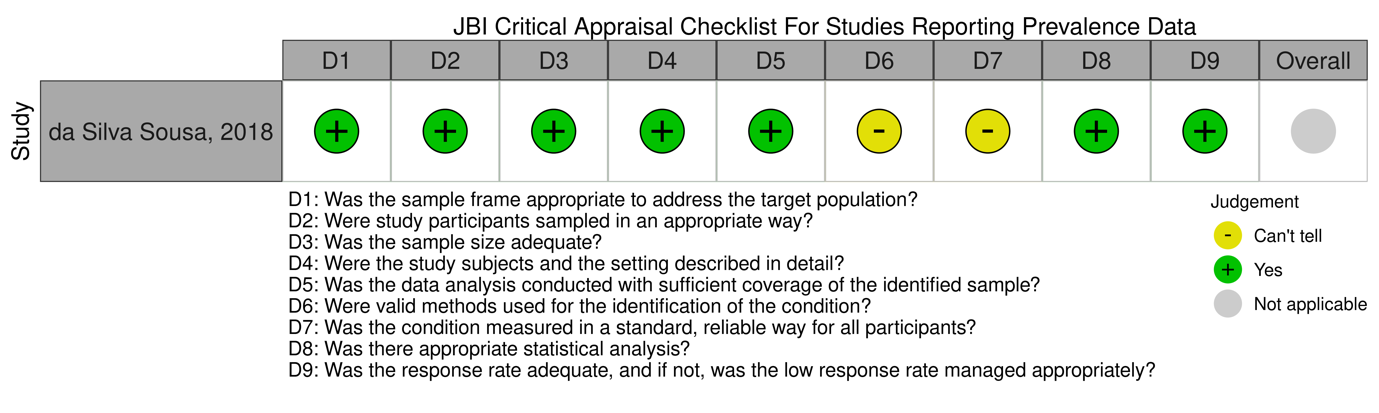


Figure C4: Critical appraisal of qualitative studies (McGuinness & Higgins, 2020)


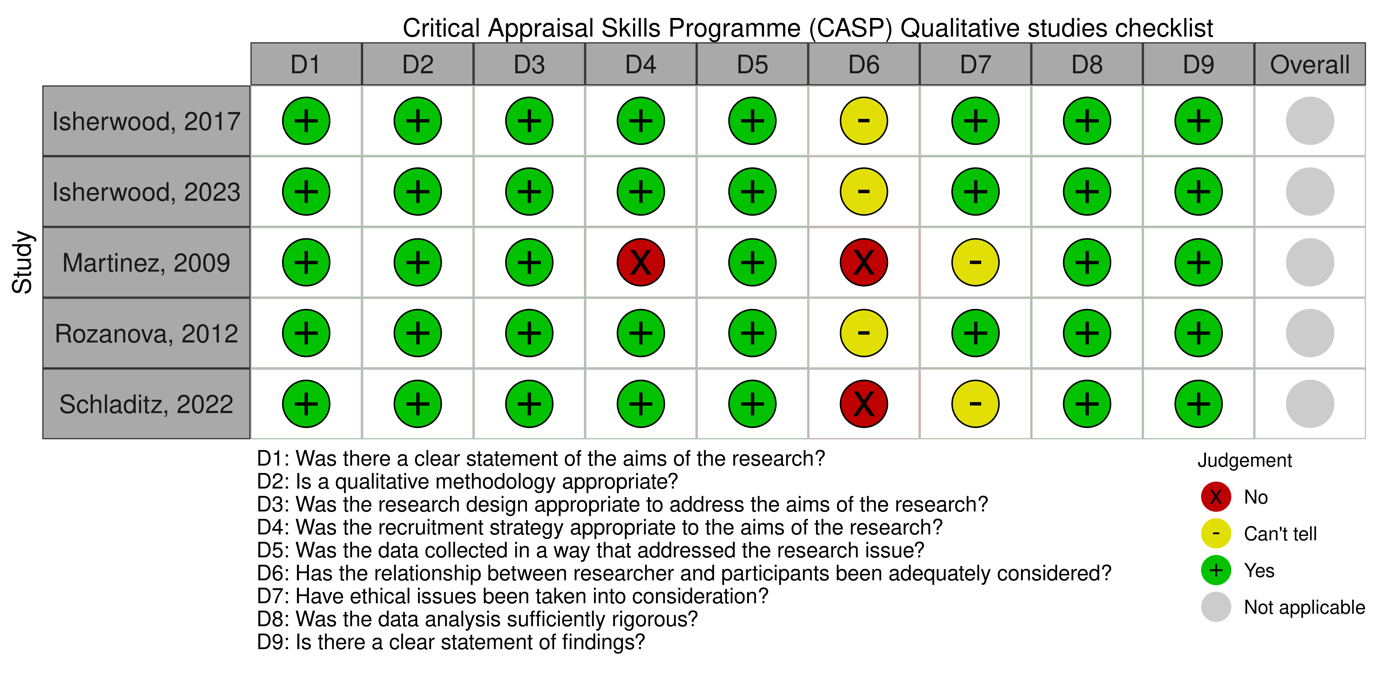


Figure C5: Critical appraisal of mixed method studies (McGuinness & Higgins, 2020)


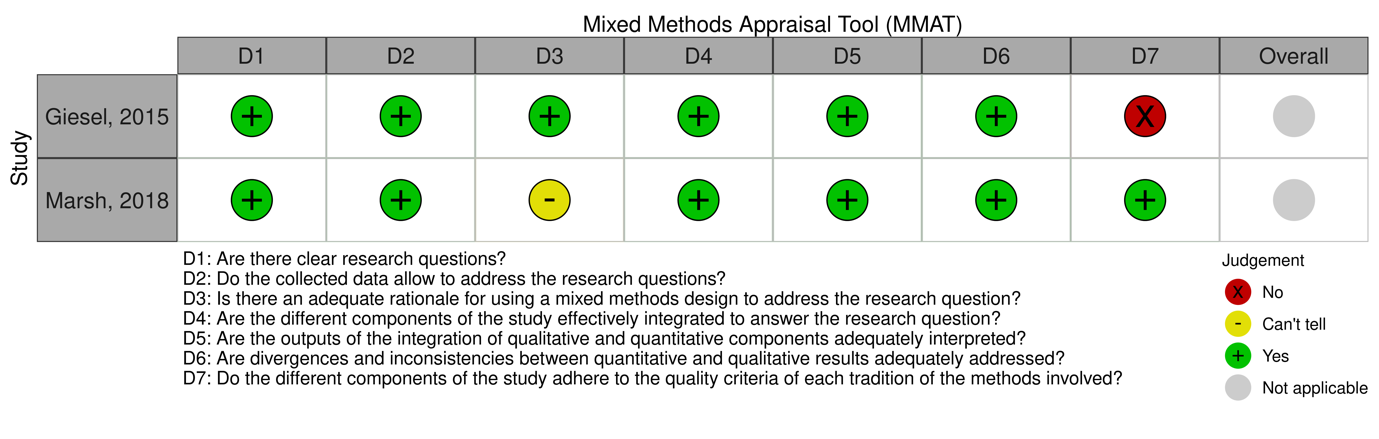


McGuinness, L. A., & Higgins, J. P. T. (2020, 2020/04/26). *Risk-of-bias VISualization (robvis): An R package and Shiny web app for visualizing risk-of-bias assessments*. John Wiley & Sons. Retrieved 2020 from <https://doi.org/10.1002/jrsm.1411>
